# Evaluation of Nonspecific Low Back Pain Using a New Detailed Visual Analogue Scale for Patients in Motion, Standing, and Sitting: Characterizing Nonspecific Low Back Pain in Elderly Patients

**DOI:** 10.1155/2012/680496

**Published:** 2012-11-18

**Authors:** Yasuchika Aoki, Shiro Sugiura, Koichi Nakagawa, Arata Nakajima, Hiroshi Takahashi, Seiji Ohtori, Kazuhisa Takahashi, Satoru Nishikawa

**Affiliations:** ^1^Department of Orthopaedic Surgery, Sakura Medical Center, Toho University, 564-1 Shimoshizu, Sakura, Chiba 285-8741, Japan; ^2^Nishikawa Orthopedics Clinic, 1-14-2 Ohsakidai, Sakura, Chiba 285-0817, Japan; ^3^Department of Orthopaedic Surgery, Graduate School of Medicine, Chiba University, 1-8-1 Inohana, Chuoku, Chiba, Chiba City 260-8670, Japan

## Abstract

Because we have a clinical impression that elderly patients have low back pain while in motion and standing, but less pain when sitting, we investigate characteristics of nonspecific low back pain (NSLBP), using a new detailed visual analog scale (VAS) scoring system. One hundred eighty-nine patients with NSLBP were divided into an elderly group (≥65 years old, *n* = 56) and a young group (<65 years old, *n* = 133). Low back pain was evaluated by a traditional VAS scoring system, the Oswestry Disability Index (ODI), and a new detailed VAS scoring system in which pain is independently evaluated in three different postural situations (in motion, standing, and sitting). No significant differences were observed in traditional VAS and ODI scores between the two groups. The results of the detailed VAS showed no significant differences between the two groups while in motion and standing. However, the elderly group showed significantly lower VAS score while sitting compared to the young group. In this study of the first use of a new detailed VAS scoring system, differences in characteristics of NSLBP between elderly and young patients were successfully detected. This minor modification of the traditional VAS may be useful for characterizing and evaluating low back pain.

## 1. Introduction

Low back pain is a common clinical problem and a significant socioeconomic problem. Although the lifetime prevalence of back pain is 60–80% [1], little is known of its pathophysiology. Clinically, the natural course of low back pain is usually favorable; acute low back pain frequently disappears within one to two weeks. In some cases, however, acute low back pain becomes chronic and quite difficult to treat and has a major socioeconomic impact. Any of the spinal structures, including intervertebral discs, facet joints, vertebral bodies, ligaments, or muscles could be an origin of back pain, which is, unfortunately, quite difficult to determine [2]. In those cases in which the origin of back pain cannot be determined, the diagnosis given is nonspecific low back pain [2].

Nonspecific low back pain is defined as low back pain not attributable to a recognizable, known specific pathology, such as infection, tumor, osteoporosis, fracture, structural deformity, inflammatory disorder, radicular syndrome, or cauda equina syndrome [2]. The intensity of low back pain is usually evaluated by a visual analogue scale (VAS), numerical rating scale, or a disability scoring system, such as the Oswestry Disability Index (ODI), Roland Morris Disability Questionnaire, and others. However, the use of these established rating systems does not fully evaluate the characteristics of low back pain. 

Previous studies suggested that low back pain varies in different situations [3, 4]. Some patients have pain in motion, but no pain when standing, and some have pain after standing for long time, but no pain in motion. O'Sullivan proposed a mechanism-based classification system dividing nonspecific low back pain into three subtypes based on pain provocative spinal postures and movement patterns [3, 4]. This classification system uses three subtypes: flexion pattern, active-extension pattern, and multidirectional pattern. In adolescents with nonspecific low back pain, sitting is a common aggravating factor and accounts for significant disability [5]. Conversely, we have clinical impression that elderly patients have low back pain while in motion and standing, but less pain when sitting.

In our institution, we have used the traditional VAS and ODI to evaluate low back pain; however, those are usually unable to detect the difference between elderly and young patients. To improve evaluation of low back pain, we established a detailed VAS scoring system, which independently evaluates the low back pain of a patient while in motion, standing, and sitting. We hypothesized that the detailed VAS may detect the difference between elderly and young patients with nonspecific low back pain. In this study, we used our new detailed VAS scoring system, together with more traditional methods, to evaluate patients with nonspecific low back pain.

## 2. Patients and Methods

**Figure 1:**
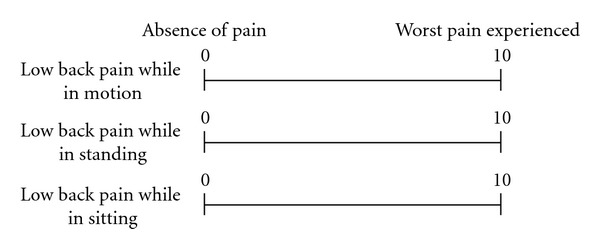
A new visual analogue scale (VAS, 0–10 cm) was developed for this study to provide a detailed evaluation of low back pain. In this new detailed VAS, low back pain is scored independently while the patient is engaged in three different postural situations: motion, standing, and sitting.

One hundred eighty-nine patients who had suffered from back pain for at least three months were included. All patients were examined by plain radiography of the lumbar spine and showed no obvious pathological finding to explain the origin of their back pain. Patients who showed any indication of such pathological problems as vertebral fractures, tumors, or infectious diseases were excluded. To exclude the possibility of radicular back pain, patients with lower extremity symptoms were excluded. Patients under 20 years old were also excluded. Low back pain was evaluated using a VAS (0–10 cm) to evaluate the patient's worst pain during the week just passed and the ODI. In addition, an VAS for low back pain in three different situations, which we established for this study, was used for a detailed evaluation. The detailed VAS evaluates low back pain while the patient is in motion, standing, and sitting (Figure 1). To establish the efficacy of this method, we performed a comparative study to examine the differences in VAS for low back pain, ODI, and the new detailed VAS between elderly patients (elderly group: ≥65 years old, *n* = 56) and younger patients (young group: <65 years old, *n* = 133). The study protocol was approved by the institutional ethics committee. 

Statistical analyses between the two groups used the unpaired *t*-test for VAS comparison, and the Mann-Whitney U test for ODI comparison. Analyses among the VASs in the three situations in each group used the repeated ANOVA. Probability values <0.05 were considered significant. Values are expressed as the mean ± standard deviation.

## 3. Results

No patient in the elderly group (mean age: 72.5 years old, range: 65–88 years old: 30 males and 26 females) or the young group (mean age: 46.0 years old, range: 20–64 years old: 59 males and 74 females) was diagnosed with a specific pathologic condition in the lumbar spine or showed lower extremity symptoms. 

**Figure 2:**
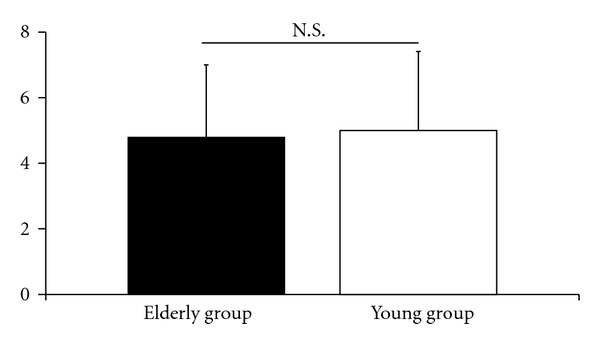
Mean scores for intensity of low back pain in patients with nonspecific low back pain scored using a traditional visual analogue scale (VAS, 0–10 cm) to evaluate the patient's worst pain during the week just passed in the elderly group (≥65 years old, *n* = 56) and young group (<65 years old, *n* = 133). Error bars represent the standard deviation. N.S.: no significant difference.

**Figure 3:**
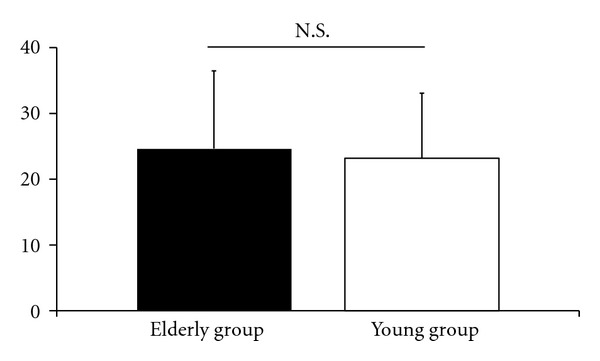
Mean scores for the Oswestry Disability Index (ODI) in patients with nonspecific low back pain in the elderly group and young group. Error bars represent the standard deviation. N.S.: no significant difference.

**Figure 4:**
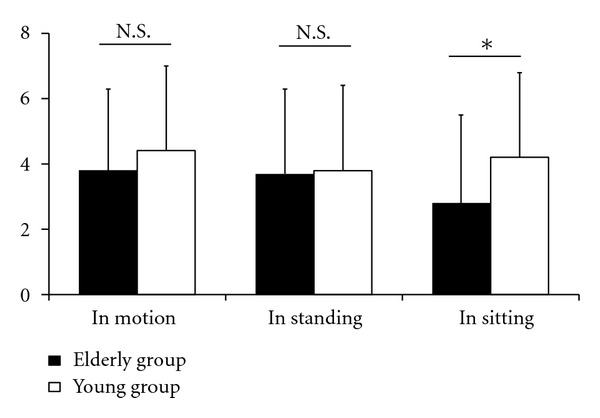
Mean scores for intensity of low back pain in patients with nonspecific low back pain scored using a new detailed visual analogue scale (VAS, 0–10 cm). Low back pain was scored while in motion, standing, and sitting in the elderly group and young group. Error bars represent the standard deviation. *P < 0.05; N.S.: no significant difference.

Mean scores for the traditional VAS for low back pain were 4.8 ± 2.2 cm in the elderly group and 5.0 ± 2.4 cm in the young group (Figure 2). No significant difference was found between the two groups. Mean scores for ODI were 24.6 ± 11.9 in the elderly group and 23.2 ± 9.8 in the young group (Figure 3) with no significant difference found between the groups. Mean scores for the detailed VAS for low back pain in the elderly group were 3.8 ± 2.5 cm in motion, 3.7 ± 2.6 cm in standing, and 2.8 ± 2.7 cm in sitting, and in the young group, 4.4 ± 2.6 cm in motion, 3.8 ± 2.6 cm in standing, and 4.2 ± 2.6 cm in sitting (Figure 4). In each group, no significant difference was observed among the VASs in the three situations. In the elderly group, the lowest intensity of low back pain was observed while sitting, with the VAS for low back pain when sitting being significantly lower than that for the young group (*P* < 0.01). 

## 4. Discussion

To date, there has been no scale measuring the intensity of low back pain in different situations used and reported in the literature. As O'Sullivan reported, nonspecific low back pain can be subclassified into flexion, active-extension, and multidirectional patterns [3, 4], thus indicating that intensity of low back pain varies depending on posture. Nachemson reported that intradiscal pressure is higher in sitting than when standing [6]. Callaghan and McGill reported differences in erector spinae muscle activity during standing and sitting [7]. In adolescents with low back pain, sitting is a common aggravating factor [5]. A recent study reported that nearly all adolescents with nonspecific low back pain described the sitting posture as the most prevalent aggravating factor [8]. There is also a report that sitting is highly associated with low back pain in young employees, aged 17–40 years old, working in a clothing factory [9]. These findings indicate that young patients feel low back pain most intensely in the sitting position.

Our study also shows that young patients showed higher VAS scores while sitting than did elderly patients, although young patients showed similar VAS scores in all three situations. The elderly group showed low VAS scores when sitting, indicating that a characteristic of nonspecific low back pain in elderly patients may be less pain in a sitting posture. In this study of the first use of the detailed VAS in a clinical setting, differences in nonspecific low back pain between elderly and young patients were successfully detected. This finding clearly supports our clinical impression that elderly patients have low back pain while in motion and standing, but less pain when sitting. 

There are no previous studies showing such clear characterization of nonspecific low back pain in elderly patients. While it is impossible to characterize low back pain using the traditional VAS evaluation, our detailed VAS will help clinicians characterize a patient's low back pain. 

Disc degeneration, one of the age-related changes of the disc structure, is thought to be a cause of chronic discogenic low back pain [10]. During the aging process, degenerated discs exhibit multiple changes, such as loss of proteoglycan content [11] and failure of nutrient supply [12, 13]. However, disc degeneration is commonly observed in patients in the absence of low back pain, suggesting that the correlation between disc degeneration and pain is not clear-cut [14–16]. Recently, Laplante et al. reported that the probability of discogenic low back pain decreases with age, while the probability of facet joint pain increases with age [17]. Clinically, it is quite difficult to determine the origin of low back pain in patients with low back pain. However, difference in pain source may explain the difference in characteristics of low back pain between elderly and young patients.

To evaluate chronic pain states, Dones et al. proposed a two-dimensional VAS system assessing both intensity and frequency [18]. These authors reported the usefulness of their VAS system for evaluating patients with trigeminal neuralgia, Arnold's neuralgia, and cluster headache [18]. With nonspecific low back pain, the intensity of pain has been shown to vary depending on posture and situations [2–4]. Thus, the frequency of pain will vary depending on the type and level of daily activity. These findings led us to evaluate low back pain patients using a detailed VAS in three different postural situations. In consideration of our findings that no significant differences were detected using the traditional VAS evaluation and ODI, our detailed VAS may be useful not only to characterize low back pain, but also to examine treatment effects of low back pain in clinical situations. For example, we observed that some patients whose traditional VAS scores for low back pain did not change after spinal surgery were nonetheless satisfied with their level of reduction of low back pain. In such cases, there is a possibility that one or two of the detailed VAS scores in the three situations decreased following surgery.

## 5. Conclusions

In the present study, our new detailed VAS could detect the difference between elderly and young patients with nonspecific low back pain, while the difference could not be detected by the traditional VAS and ODI. Thus, we believe our hypothesis is correct, and this minor modification of the VAS will not only be useful and easy to use for scoring patients with low back pain, but may help clinicians treating patients with low back disorders.
